# Introducing neurofilament light chain measure in psychiatry: current evidence, opportunities, and pitfalls

**DOI:** 10.1038/s41380-024-02524-6

**Published:** 2024-03-19

**Authors:** Francesco Bavato, Christian Barro, Laura K. Schnider, Joel Simrén, Henrik Zetterberg, Erich Seifritz, Boris B. Quednow

**Affiliations:** 1 Experimental and Clinical Pharmacopsychology, Department of Psychiatry, Psychotherapy and Psychosomatics; Psychiatric University Hospital Zurich, University of Zurich, Zurich, Switzerland; 2 Ann Romney Center for Neurologic Diseases, Department of Neurology, Brigham and Women’s Hospital, Harvard Medical School, Boston, MA USA; 3 Department of Psychiatry and Neurochemistry, Institute of Neuroscience and Physiology, The Sahlgrenska Academy at the University of Gothenburg, Mölndal, Sweden; 4 Clinical Neurochemistry Laboratory, Sahlgrenska University Hospital, Mölndal, Sweden; 5 Department of Neurodegenerative Disease, UCL Institute of Neurology, Queen Square, London UK; 6 UK Dementia Research Institute at UCL, London, UK; 7 Hong Kong Center for Neurodegenerative Diseases, Clear Water Bay, Hong Kong, China; 8 Wisconsin Alzheimer’s Disease Research Center, University of Wisconsin School of Medicine and Public Health, University of Wisconsin-Madison, Madison, WI USA; 9 Department of Psychiatry, Psychotherapy and Psychosomatics; Psychiatric University Hospital Zurich, University of Zurich, Zurich, Switzerland; 10 Neuroscience Center Zurich, University of Zurich and Swiss Federal Institute of Technology Zurich, Zurich, Switzerland

**Keywords:** Biological techniques, Diagnostic markers

## Abstract

The recent introduction of new-generation immunoassay methods allows the reliable quantification of structural brain markers in peripheral matrices. Neurofilament light chain (NfL), a neuron-specific cytoskeletal component released in extracellular matrices after neuroaxonal impairment, is considered a promising blood marker of active brain pathology. Given its sensitivity to a wide range of neuropathological alterations, NfL has been suggested for the use in clinical practice as a highly sensitive, but unspecific tool to quantify active brain pathology. While large efforts have been put in characterizing its clinical profile in many neurological conditions, NfL has received far less attention as a potential biomarker in major psychiatric disorders. Therefore, we briefly introduce NfL as a marker of neuroaxonal injury, systematically review recent findings on cerebrospinal fluid and blood NfL levels in patients with primary psychiatric conditions and highlight the opportunities and pitfalls. Current evidence suggests an elevation of blood NfL levels in patients with major depression, bipolar disorder, psychotic disorders, anorexia nervosa, and substance use disorders compared to physiological states. However, blood NfL levels strongly vary across diagnostic entities, clinical stage, and patient subgroups, and are influenced by several demographic, clinical, and analytical factors, which require accurate characterization. Potential clinical applications of NfL measure in psychiatry are seen in diagnostic and prognostic algorithms, to exclude neurodegenerative disease, in the assessment of brain toxicity for different pharmacological compounds, and in the longitudinal monitoring of treatment response. The high inter-individual variability of NfL levels and the lack of neurobiological understanding of its release are some of the main current limitations. Overall, this primer aims to introduce researchers and clinicians to NfL measure in the psychiatric field and to provide a conceptual framework for future research directions.

## Introduction

In several neuroimaging studies, almost every psychiatric disorder has been associated with a wide range of structural brain alterations, including both white and gray matter structures [1–5]. However, imaging markers to quantify and monitor brain pathology in clinical psychiatric settings are still lacking, making the introduction of new diagnostic tools still urgently needed [6].

**Fig. 1:**
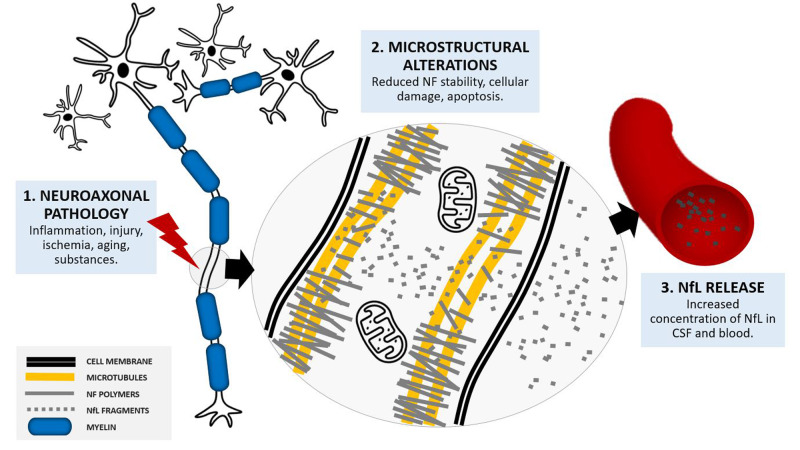
NfL release into extracellular fluids.

An intriguing approach to assess the integrity of brain structures with a minimally invasive procedure consists in detecting the levels of specific brain proteins in extracellular matrices, such as cerebrospinal fluid (CSF) or blood. In particular, neurofilaments are emerging as the most promising blood markers of neuroaxonal pathology [7]. Neurofilaments are cytoskeletal components, predominantly expressed in long myelinated axons and thought to support axonal stability and high-velocity nerve conduction [8]. In pathological processes that involve axonal integrity, neurofilaments are released into the CSF and, in smaller amount, into the peripheral blood (Fig. 1). Despite a clear understanding of the underlying cellular processes is still lacking, neurofilament release into peripheral fluids was shown to be proportional to the amount of active brain pathology in a number of neurological conditions [7]. Changes in neurofilament levels can even be detected in presence of subclinical damage such as asymptomatic strokes or white matter hyperintensities linked to silent cerebrovascular disease [9, 10]. Consequently, over the past few years an increasing number of research studies have assessed blood concentrations of neurofilaments – especially of neurofilament light chain (NfL), the smallest and most abundant neurofilament subunit – in different clinical conditions. Promising findings in inflammatory, neurodegenerative, traumatic, and cerebrovascular diseases suggest a clinical application of NfL as an unspecific, all-around tool to assess the extent of brain damage [11–15]. In accordance with these findings, NfL has been proposed as “troponin for the brain” [16]. NfL measure in neurological conditions may allow: (1) to detect early pathological processes [9], (2) to quantify the degree of active brain pathology [17], (3) to monitor treatment response [11], and (4) to predict clinical outcome [18].

The association of NfL with aging and cognitive functioning in healthy individuals also suggests a high sensitivity of NfL levels in the detection of microstructural alterations at a subclinical level, thus hinting at potential sensitivity for neuropathology at a magnitude expectable in psychiatric conditions [19, 20]. Some investigations in affective disorders [21, 22], schizophrenia [23], substance use disorders (SUD) [24, 25], and anorexia nervosa (AN) [26], reported alterations of NfL levels in either CSF, blood, or both, and with magnitudes intermediate between physiological states and some neurological disorders with current disease activity (e.g., frontotemporal dementia [FTD], Parkinson’s disease [PD], or multiple sclerosis [MS] with radiological active status) [27]. Nonetheless, the research focus on NfL in psychiatric conditions has been initially limited in the differentiation from neurological conditions, and further clinical implications in psychiatry have been neglected so far [28, 29]. Therefore, considering the introduction of NfL in the neurological practice, it is now crucial to define how psychiatric conditions are related to blood NfL levels, which opportunities are offered, and which pitfalls should be considered for NfL application in psychiatric conditions.

The scope of this review is to provide researchers and clinicians with a primer on NfL as a blood-based marker for neuroaxonal pathology in psychiatry and to guide future research on the field.

## NfL as a blood biomarker

### Structure and function

Neurofilaments are intermediate filament and class IV proteins including following subunits: neurofilament heavy chain (NfH, 200–220 kDa), medium chain (NfM, 145–160 kDa), light chain (NfL, 68–70 kDa), and α-internexin (58–66 kDa, in the central nervous system) or peripherin (57-59 kDa, in the peripheral nervous system) [8]. NfL, the most abundant and most soluble of neurofilaments, is transcribed from the *Neurofilament Light Polypeptide* (*NEFL*) gene located on chromosome 8 (8p21.2). Together with NfM and NfH proteins, NfL assembles into linear hetero-polymers [30]. Under physiological conditions, neurofilament polymers show high stability and slow turnover in the cytoplasm of mature neurons [31]. Regarding its functions, NfL is known to support the radial expansion of large myelinated axons, which explains mutations in the *NEFL* gene resulting in nerve damage as in Charcot-Marie-Tooth disease [32]. In the central nervous system, NfL subunits have also been found in synaptic terminals in oligomeric form, inferring a structural or modulatory role of NfL in synaptic level [33]. In this direction, *NEFL* deletion was shown to heavily interfere with dendritic spine morphology, NMDAR-GluN1 expression, and synaptic plasticity [34]. For a detailed description of the neurobiology of neurofilaments (e.g., transport, phosphorylation, degradation, clearance), we would like to refer to the extensive work by Yuan and colleagues [8, 35].

### Immunoassay methods and pre-analytical/analytical variables

The qualitative detection of NfL in CSF was first possible using immunoblotting techniques. Second-generation enzyme-linked immunosorbent assays (ELISAs) later allowed to quantitatively measure CSF NfL levels while third-generation electrochemiluminescence (ECL) technology made possible to detect NfL in blood [36]. However, it was the development of fourth-generation immunoassay, such as Single Molecule Array (SIMOA, Quanterix) and microfluidic cartridge-based automatized immunoassay platforms (ELLA, ProteinSimple) that enabled the reliable quantification of NfL in blood serum and plasma [36, 37]. Importantly, the introduction of fully automated fourth-generation platforms (i.e., SIMOA by Quanterix and ELLA by Bioteche) and the development of NfL assays for clinical chemistry analyzers newly offer high reproducibility and multi-center validation, thus, making NfL suitable for clinical use [38, 39]. A more detailed discussion on the next steps required to move NfL measure into clinical routine can be found in previous works [39, 40]. In general, good stability to pre-analytical and analytical conditions have been reported for blood NfL, with limited effects reported for multiple freeze-thawing-cycles and prolonged exposure to room temperature [40, 41]. NfL concentrations were shown to be strongly correlated between EDTA plasma and serum, despite plasma levels being around 20% lower [41]. The performance of both fourth-generation assays available (SIMOA and ELLA) have been demonstrated to be comparable [37].

### NfL levels in blood versus CSF and the role of the blood–brain barrier

Strong correlations have been shown between NfL levels in blood and CSF, with CSF levels being around 40-fold higher compared to blood [42]. These findings support the use of blood NfL as a reliable surrogate measure of CSF NfL but some concerns remain. In particular, the degree of permeability of the blood–brain barrier (BBB) and blood–CSF barrier to NfL levels is not fully elucidated [43]. It is still possible that neuropsychiatric conditions might be associate with a disrupted BBB, and, thus, leak more NfL from the CSF into the blood. Studies addressing potential associations of NfL levels with the ratio between CSF and serum albumin (a proxy marker of BBB permeability) reported contrasting findings [41, 44]. Here, the strong collinearity between brain pathology and BBB disruption in some clinical conditions might hinder a clear separation of the two processes in vivo studies [45]. Tailored studies including direct or indirect measures of BBB permeability together with NfL measurement in primary psychiatric disorders are also lacking. Notably, an animal model using cranial irradiation in mice found no timely correlation between serum NfL concentration and BBB permeability [46]. Overall, very little evidence supports an effect of BBB permeability on blood NfL levels but further investigations are required.

### Biological fluctuations, biorhythmic effects, and half-life

The degree of intra-individual variation between NfL measurements taken in close temporal succession (hours to days) in healthy individuals (in absence of clinically relevant biological events) has been demonstrated to be small [47, 48]. The mean coefficient of variation of NfL levels between repeated measures have been calculated to be around 7.4% [48]. Thus, NfL stability is a significant advantage for longitudinal studies compared to other brain-derived markers (e.g., brain-derived neurotrophic factor, BDNF) [47, 49]. In a recent study by Hviid and colleagues, the semidiurnal variations of blood NfL levels were measured at a same experimental day (9 AM, 12 PM, 3 PM, 6 PM, 9 PM) [47]. In this study, no significant difference in NfL levels was reported across time points. Similarly, Benedict et al. reported no significant evening to morning changes of NfL levels after overnight sleep loss or normal nightly sleep [50]. Overall, there is no evidence suggesting the presence of biorhythmic alterations of NfL levels, and the time of sampling is not required to be considered as a confounding variable [40, 41]. In the absence of experimental validation on biological half-life, the virtual half-life of NfL levels in blood as calculated by kinetic models is described to be around 500–1000 h [51, 52]. However, this estimation is based on longitudinal data on NfL release and normalization after a traumatic brain injury (TBI). The elevation of blood NfL levels days after a TBI might be driven by subsequent reorganization in the brain and not only confined to the acute release after the head impact. Similarly, the slow normalization of NfL levels over months observed in patients with MS under disease-modifying treatments might be related to the slow reduction of brain pathology rather than to kinetic effects of blood NfL [11]. While fine-grained investigation of NfL dynamic in other conditions are limited, indirect evidence from individuals with epileptic seizures might suggest that the biological half-life could be much shorter. Further investigations including repeated NfL measurements in different clinical conditions are still required to draw final conclusions on the kinetic of blood NfL levels.

### Physiological factors and reference values

**Fig. 2:**
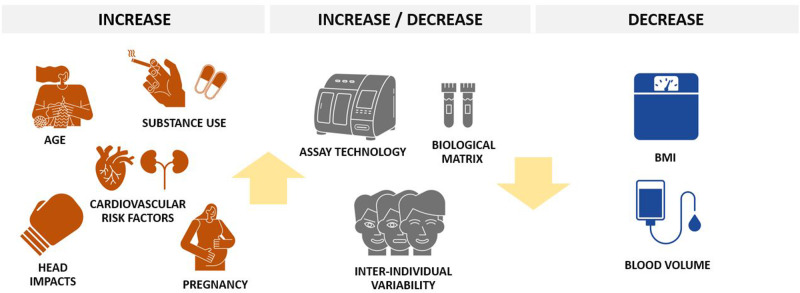
Main established physiological, clinical and analytical confounding factors for NfL measure in blood.

Several physiological factors might influence NfL levels and could at least partially explain the high degree of inter-individual variability of blood NfL levels that has been reported in healthy individuals (see Fig. 2). First, a large body of evidence demonstrated a nonlinear, age-dependent increase of NfL levels that has been estimated at around 1–2% per year in early and middle adulthood and might reach 4–5% per year in late adulthood [9, 18]. In healthy adults, NfL levels are positively associated with volumetric brain measures and white matter alterations [9, 53]. An age-dependent increase in the inter-individual variance of NfL levels have also been reported, with higher NfL levels predicting future volumetric brain loss [9, 54]. The association between NfL levels and age-dependent cognitive decline in elderly adults further support its role as a marker of brain aging [9, 19, 55]. On the contrary, during childhood and adolescence NfL levels were found to decrease by an estimated 6–8% per year until the age of 10 and to be mostly stable up to the age of 22 years [56]. Accordingly, most studies suggest the introduction of age-dependent reference values for blood NfL [56, 57]. Blood volume and body mass index (BMI) also represent main confounding variables, showing an inverse correlation with blood NfL levels, probably driven by dilution effects. Of note, the magnitude of the effect of BMI on NfL levels in childhood is only marginal [56]. Online tools to calculate z-scores and percentiles for blood NfL levels after correction for age and BMI are now available [57–59]. When correcting for age and BMI, no effects of ethnicity on blood NfL levels have been shown in healthy individuals to date [41]. Despite early reports of sex differences on NfL levels, larger studies demonstrated no effect of sex on blood NfL in healthy population when age- and BMI-corrected [9, 60]. Potential interactions of sex and ethnicity on NfL levels in specific clinical conditions might still be observed [55, 61]. In pregnancy, an increase in blood NfL levels have been reported, despite the pregnancy-related expansion of blood volume should lead to dilution and therefore decrease in NfL levels [28]. An increasing amount of NfL derived from the growing nervous system of the fetus or structural remodeling of the mother’s brain have been discussed as potential explanations [28, 41].

### Clinical confounding factors

Some clinical factors might potentially influence NfL levels beyond primary pathologies of the central nervous system and should be considered when measuring NfL levels in psychiatric conditions (see Fig. 2). Cardiovascular risk factors such as the levels of glycosylated hemoglobin (HbA1c), systolic blood pressure, smoking, and dyslipidemia, have been associated with an increase of NfL levels [28]. Hence, some of these effects may be driven by subclinical cerebrovascular involvement resulting in subtle neuroaxonal pathology [41]. Alterations of renal function (decrease of glomerular filtration rate) has been shown to be associated with increased blood NfL levels, although the association is mainly manifest in participants with chronic kidney disease [58, 62]. The influence of chronic substance use (e.g., alcohol, cocaine, ketamine) on NfL levels has been demonstrated in patients with SUD [24, 25]. However, the potential impact of occasional substance use on NfL levels in individuals without SUD has been barely investigated so far [63, 64].

It is important to notice that NfL is also expressed in the peripheral nervous system and that blood NfL elevation has been reported in both demyelinating and axonal forms of peripheral neuropathy [65, 66]. Therefore, the presence of peripheral pathology should also be considered as a potential confounding factor in the investigation of NfL levels. Finally, increased CSF and blood NfL levels have been consistently associated with accidental head impacts and with sport-related head concussion in football, ice hockey, and boxing with different degrees of brain injury and even in absence of clinical presentation [14, 67].

### Conceptual framework for clinical application in neurological disorders

NfL has been suggested as an unspecific, all-around marker to be implemented in neurological disorders following the conceptual framework of troponin in cardiology and C-reactive protein in immunology [16, 68]. Similar to troponin and C-reactive protein, NfL release is not informative of a specific etiological process (i.e., inflammatory, ischemic, degenerative, or traumatic), neither is it uniquely associated with specific neuroimaging alterations (i.e., gray vs. white matter, cortical vs. subcortical areas). Indeed, NfL levels were found to be elevated in most neurological condition. Therefore, the clinical applications of NfL measure relates to the ability of quantifying active brain pathology independently of the underlying etiology, rather than increasing diagnostic specificity [41]. In neuroinflammatory disorders such as MS, NfL levels were found to increase with disease relapses or new MRI lesions and to decrease with effective treatments [11, 42, 69]. The normalization of NfL levels observed under disease modifying therapies in MS also make it an ideal tool to monitor treatment response in both experimental studies and clinical settings [70, 71]. Here, NfL levels might early detect treatment responsiveness and guide clinical decisions without waiting for disease relapses.

In contact sports, mild TBI are considered a silent epidemic leading to increased risk of neurological deficits when athletes return to play prematurely after an head concussion [72]. In this context, NfL measure was found to be a helpful instrument to guide the return-to-play decision in the follow-up of a head concussion by detecting the persistence or resolution of brain pathology [14].

NfL levels are also sensitive to subclinical neurodegeneration, with NfL increase in neurodegenerative disorders such as Alzheimer’s disease (AD) already detectable years prior to clinical onset in both early and late onset AD [73, 74]. Thus, NfL measure could be used in memory clinic settings to allow early identification of neurodegenerative disorders and to discriminate them from non-degenerative forms of cognitive decline [15, 75].

## NfL in psychiatric disorders: current evidence

### Search strategy

**Fig. 3:**
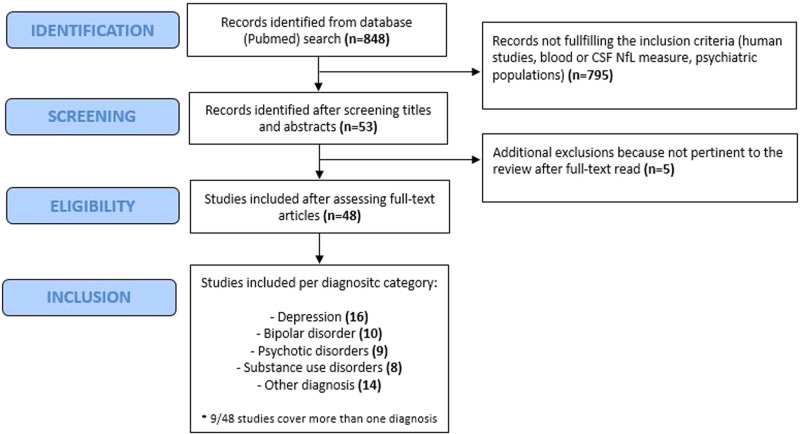
Flow chart of systematic review.

The literature search on current evidence followed the Preferred Reporting Items for Systematic Reviews and Meta-Analysis (PRISMA) guidelines for systematic reviews. Two reviewers (FB, LS) systematically searched PubMed for studies reporting on NfL in primary psychiatric disorders (PPD) from last database opening on 11th August 2023. The search strategy was performed using specific search terms (i.e., [NfL OR neurofilament light chain] AND [human] AND [psychiatry OR psychiatric OR depression OR depressive OR schizophrenia OR psychosis OR substance use disorder OR anorexia OR bipolar disorder]). Titles and abstracts were screened to select articles relevant to the purpose of the review. Their reference lists were also hand-searched to increase the identification of useful data. We selected studies assessing blood and/or CSF NfL levels in patients with PPD. The selection was shared among all co-authors. Only studies on humans and articles in English language were included. From the *n* = 848 records screened, *n* = 47 were included after assessing full-text articles. See flow chart Fig. 3. Studies included in the final selection are summarized in Tables 1–5. Studies of NfL in patients with psychiatric symptoms due to neurological, inherited or systemic conditions were excluded by the current selection.

**Table 1:** Studies investigating NfL levels in patients with depression.

Clinical characteristics	Matrix	Assay	N	Age	Comparison with HC	Other findings	Confounders considered	Reference
Depression with/without psychosis, organic depression included	Serum	SIMOA	28	52	≈	Lower NfL levels in depression compared to FTD	Yes (age)	Al Shweiki et al. [85]
MDD, current episode	Serum	ELISA	94	38	↑	_	Yes (age, sex, BMI)	Al-Hakeim et al.
Depression, unspecified	Plasma	SIMOA	37	32	≈	Lower NfL in depression compared to neurodegenerative disorders; abnormal NfL levels in 12% of patients with depression	Partially (age)	Ashton et al. [27]
MDD, severe episodes excluded	Serum	HRP-ELISA	24	31	↑	–	Yes (age, sex, BMI)	Bai et al. [90]
Clinically stable MDD, current episode	Serum	SIMOA	44	34	↑	Negative association of NfL with cognitive processing speed (DSST)	Yes (age, sex, BMI)	Bavato et al. [21]
MDD with moderate to severe episodes, treatment resistant	Serum	SIMOA	15	49	≈	No effect of ECT on NfL (before first ECT vs. after last ECT)	Partially (age, sex)	Besse et al. [95]
MDD with current episode, 80% first episode	Plasma	HRP-ELISA	40	28	↑	Negative association of NfL with executive functions (WCST)	Yes (age, sex, BMI)	Chen et al. [87, 110]
MDD diagnosis but unspecified clinical state	CSF	SIMOA	9	51	≈	Lower NfL levels in MDD compared to FTD	Yes (age, sex)	Eratne et al. [68]
MDD diagnosis but unspecified clinical state	CSF	ELISA	16	54	≈	Lower NfL levels in MDD compared to FTD	Yes (age)	Eratne et al.
Geriatric depression, only women	CSF	ELISA	11	74	↑	–	Yes (age)	Gudmundsson et al. [86]
MDD under stable treatment, 53% comorbid KD	Serum	SIMOA	65	34	( ↑ )	Eleveted NfL levels in MDD with KD but not in MDD alone	Yes (age, sex, BMI, smoking)	Huang et al. [93]
MDD with mild to severe episodes, no current antidepressant treatment	Serum	SIMOA	110	42	≈	No association of NfL with cogntive scores; NfL increase at follow-up	Yes (age, sex, BMI)	Hviid et al. [94]
MDD, first episode, medication-naïve	Serum	ELISA	82	34	↑	Positively correlation of NfL levels with depressive scores and white matter alterations	Partially (age)	Jiang et al. [91]
MDD, treatment resistant	Plasma	HRP-ELISA	71	48	↑	NfL predicted treatment response for ketamine IV treatment	Yes (age, sex, BMI)	Lin et al. [92]
MDD with moderate to severe episodes, with/without psychotic symptoms	Serum	SIMOA	45	48	?	No diagnostic specificity for NfL; comparison HC vs. MDD not reported	Partially (age)	Steinacker et al. [88]
MDD, treatment resistant	CSF	ELISA	9	58	none	No effect of ECT on NfL (before first ECT vs. after last ECT)	No	Zachrisson et al. [101]
MDD, unspecified clinical state	Plasma	ELISA	31	40	≈	–	Partially (age, sex)	Wallensten et al. [133]

*MDD* major depressive disorder, *KD* ketamine dependence, *HC* healthy controls, *CSF* cerebrospinal fluid, *SIMOA* single molecule array, *ELISA* enzyme-linked immunosorbent assay, *HRP* horseradish peroxidase, *FTD* frontotemporal dementia, *DSST* digit symbol substitution task, *ECT* electroconvulsive treatment, *WCST* Wisconsin card sorting test, *BMI* body mass index

**Table 2:** Studies investigating NfL levels in patients with bipolar disorder.

Clinical characteristics	Matrix	Assay	*N*	Age	Comparison with HC	Other findings	Confounders considered	Reference
BD type I, II, cyclothymia, or not otherwise specified	CSF	ELISA	133	35	↑	Positive association of NFL with antipsychotic treatment	Yes (age, sex, BMI)	Jakobsson et al. [22]
BD type I or II, current euthymic state	CSF	ELISA	82	38	↑	Association of NfL with deficits in memory and verbal functions	Yes (age, sex)	Rolstad et al. [109]
BD type I, inpatients with current depressive episode	Plasma	SIMOA	45	48	↑	Correlation between NfL and white matter alterations	Partially (age)	Aggio et al. [106]
BD with manic, depressive, or mixed episode	Serum	SIMOA	11	48	?	No diagnostic specificity for NfL, comparison HC vs. BD not reported	Partially (age)	Steinnacker et al. 2021
BD in various clinical state, severe manic or depressive episodes excluded	Serum	HRP-ELISA	25	33	↑	Inverse correlation between NfL and working memory task (2-back)	Yes (age, sex, BMI)	Bai et al. [90]
Early onset BD, current manic episode	Serum	HRP-ELISA	38	16	↑	_	No	Ceylan et al. [107]
BD type I or II, current euthymic state	CSF, Plasma	ELISA, SIMOA	85	33	≈	No longitudinal increase of NfL after an affective episode (*n* = 35)	Yes (age, sex, substance use)	Knorr et al. [108]
BD type 1, current euthymic state	Serum	SIMOA	100	47	none	Association of NfL with neurocognitive scores	Yes (age, sex)	Chen et al. [87, 110]
BP diagnosis but unspecified clinical state and subtype	CSF	ELISA	12	46	≈	Lower NfL levels in BP compared to FTD	Yes (age)	Eratne et al.
BD with current manic, depressive, or mixed episode	Serum	SIMOA	11	51	≈	Lower NfL levels in BP compared to FTD	Yes (age)	Al Shweiki et al. [85]

*BP* bipolar disorder, *HC* healthy controls, *CSF* cerebrospinal fluid, *SIMOA* single molecule array, *ELISA* enzyme-linked immunosorbent assay, *HRP* horseradish peroxidase, *FTD* frontotemporal dementia, *BMI* body mass index.

**Table 3:** Studies investigating NfL levels in patients with psychotic disorders.

Clinical characteristics	Matrix	Assay	N	Age	Comparison with HC	Other findings	Confounders considered	Reference
SZ, paranoid or undifferentiated	Serum	SIMOA	11	41	≈	Lower NfL levels in SZ compared to FTD	Yes (age)	Al Shweiki et al. [85]
SZ spectrum	CSF	ELISA	9	51	≈	Lower NfL levels in SZ compared to FTD	Yes (age, sex)	Eratne et al. [68]
SZ, treatment-resistant, under clozapine (*n* = 82), or not (*n* = 13)	Plasma	SIMOA	95	40	≈	Increased proportion of SZ patients and siblings with abnormal NfL (90^th^ percentile)	Yes (age, BMI, dyslipidaemia)	Eratne et al.
SZ, spectrum, FEP or chronic/recurrent SZ	CSF	ELISA	100	34	≈	Increased NfM in SZ compared to HC	Partially (sex, albumin)	Runge et al. [115]
SZ, clinically stable	Serum	SIMOA	44	34	≈	Increased proportion of SZ patients with abnormal NfL (95^th^, 99^th^ percentiles)	Yes (age, sex, BMI)	Bavato et al. [21]
FEP	Serum	SIMOA	45	20	( ↑ )	Cutoff of NfL ≥15 pg/mL distinguished NMDARe from FEP, comparison with HC only partially reported	Yes (age, sex)	Guasp et al. [116]
Early onset SZ	Serum	HRP-ELISA	35	16	↑	_	Partially (age, sex)	Ceylan et al. [107]
Chronic SZ or FEP	Plasma	ELISA	42	41	↑	Higher NfL levels in the SZ subgroup treated with clozapine	Partially (age, sex)	Rodrigues-Amorim et al. [23]
SZ, unclear clinical state	CSF	ELISA	17	42	≈	Lower NfL levels in SZ compared to FTD	Yes (age)	Eratne et al.

*SZ* schizophrenia, *FEP* first episode psychosis, *HC* healthy controls, *CSF* cerebrospinal fluid, *SIMOA* single molecule array, *ELISA* enzyme-linked immunosorbent assay, *HRP* horseradish peroxidase, *FTD* frontotemporal dementia, *NfM* neurofilament medium chain, *NMDARe* anti–NMDA receptor encephalitis, *BMI* body mass index.

**Table 4:** Studies investigating NfL levels in patients with substance use disorders.

Clinical characteristics	Matrix	Assay	*N*	Age	Comparison with HC	Other findings	Confounders considered	Reference
KD, treatment-seeking	Serum	SIMOA	65	32	↑	Higher NfL levels in patients with lifetime history of MDD	Yes (age, BMI, sex, smoking)	Liu et al. [25]
KD with or without comorbid MDD	Serum	SIMOA	53	33	↑	Higher NfL levels in patients with comorbid MDD and KD	Yes (age, BMI, sex, smoking status)	Huang et al. [93]
Chronic cocaine users	Plasma	SIMOA	35	33	↑	Longitudinal association between amount of cocaine use and NfL levels	Yes (age, sex, BMI)	Bavato et al. [24]
Chronic MDMA users	Serum	ELLA	39	30	≈	Absence of white matter alterations in MDMA users confirmed by DTI measures	Yes (age, sex, BMI, other substances)	Zimmermann et al. [64]
Alcohol dependence	Serum	ELISA	50	49	↑	Negative association of NfL with cognition (MoCA) and structural brain alterations	Yes (age, sex)	Li et al. [121]
Severe AUD, first day of hospitalization for withdrawal	Plasma	SIMOA	36	49	↑	Higher NfL levels in current users compared to patients with at least 3 months of abstinence	Yes (age, sex)	Clergue-Duval et al. [122]
Heavy drinking participants	Plasma	ELISA	71	44	none	Positive associations between NfL and structural brain alterations in heavy drinkers	Yes (age, sex, BMI, cannabis use)	Karoly et al. [123]
Mixed SUD (alcohol, cocaine, cannabis, sedatives, opioids)	Plasma	SIMOA	60	41	↑	Positive associations between NfL levels and cognitive dysfunctions	Yes (age)	Requena-Ocaña et al.

*SUD* substance use disorder, *AUD* alcohol use disorder, *MDD* major depressive disorder, *KD* ketamine dependence, *HC* healthy controls, *CSF* cerebrospinal fluid, *SIMOA* single molecule array, *ELISA* enzyme-linked immunosorbent assay, *HRP* horseradish peroxidase, *FTD* frontotemporal dementia, *DTI* diffusion tensor imaging, *MoCA* Montreal cognitive assessment, *BMI* body mass index.

**Table 5:** Studies investigating NfL levels in patients with other primary psychiatric disorders.

Clinical characteristics	Matrix	Assay	*N*	Age	Comparison with HC	Other findings	Confounders considered	Reference
ASD	Serum	SIMOA	83	5	↑	NfL positively correlated with ASD severity	Yes (age, sex, BMI)	He et al. [126]
ASD	Serum	ELISA	43	10	≈	NfL negatively correlated with stereotyped behavior and sensorial sensitivity	Partially (age)	Paketci et al.
ASD	Serum	SIMOA	42	7	↑	_	No	Simone et al. [125]
AN, current (*n* = 124) or recovered (*n* = 125)	Plasma	SIMOA	249	26	↑	Elevated NfL in current AN but not in recovered AN patients	Yes (age, BMI)	Nilsson et al. [26]
AN, acute	Serum	SIMOA	53	16	↑	Decrease of NfL with treatment	Yes (age, BMI)	Hellerhoff et al. [128]
AN, acute	Serum	SIMOA	52	16	↑	Association of NfL with decreased cortical thickness	Yes (age, BMI)	Hellerhoff et al. [129]
AN, assessed 30 years after diagnosis	Serum	SIMOA	34	44	↑	_	Yes (age, BMI)	Wentz et al. [130]
AN, long-term weight-recovered	Serum	SIMOA	55	22	≈	Positive association of NfL with duration of illness	Yes (age)	Doose et al. [131]
Exhaustion disorder	Plasma	SIMOA	150	44	↑	Decrease of NfL at long-term follow-up	Yes (age, sex)	Hansson et al. [132]
Exhaustion disorder	Plasma	ELISA	31	45	≈	_	Partially (age, sex)	Wallensten et al. [133]
PTSD spectrum, military veterans	Plasma	SIMOA	478	33	none	Indirect association between PTSD severity and NfL via AIM2 methylation	Yes (age, sex)	Hawn et al.
Mixed PPD (psychotic and mood disorders)	Serum	SIMOA	34	56	none	Lower NfL in psychiatric conditions compared to FTD	Yes (age)	Katisko et al. [84]
Mixed PPD (MDD, BP, SZ, anxiety disorders)	CSF	ELISA	64	65	none	Lower NfL in psychiatric conditions compared to neurodegenerative disorders	No	Fourier et al.

*ASD* autism spectrum disorder, *AN* anorexia nervosa, *PPD* primary psychiatric disorder, *BP* bipolar disorder, *SZ* schizophrenia, *MDD* major depressive disorder, *PTSD* posttraumatic stress disorder, *HC* healthy controls, *CSF* cerebrospinal fluid, *SIMOA* single molecule array, *ELISA* enzyme-linked immunosorbent assay, *HRP* horseradish peroxidase, *FTD* frontotemporal dementia, *DSST* digit symbol substitution task, *ECT* electroconvulsive treatment, *WCST* Wisconsin card sorting test, *BMI* body mass index.

### Major depressive disorder

In patients with major depressive disorder (MDD), regional volumetric reductions and widespread alterations of white matter tracts have been consistently described, with more pronounced impact observed in patients with recurrent episodes and longer illness duration [5, 76, 77]. In this regard, chronic activation of stress-related pathways has been suggested to induce neurodegeneration and accelerated brain aging in patients with MDD [78]. A potential association of depression with alterations of neurofilaments was initially reported in experimental animal models [79, 80]. Reduced hippocampal concentrations of neurofilaments in rat model of depression were shown to be restored by pharmacological (i.e. fluoxetine and amitriptyline) and non-pharmacological (i.e. enriched environment) interventions [81–83].

The first studies investigating NfL levels in patients with depression have been mainly performed in samples of elderly adults to test the diagnostic performance of NfL levels in differentiating neurodegenerative from PPD [27, 68, 84, 85]. While increased CSF NfL levels were observed in a small sample of elderly women with history of MDD (*n* = 11) [86], normal blood levels were reported in other small samples of elderly patients with MDD (range: *n* = 9-37) [27, 68, 85]. In this context, specific cut-off levels for blood NfL were shown to discriminate patients with some neurological conditions (i.e., PD, FTD) from patients with PPD (area under the curve [AUC] = 0.70–0.85), thus supporting the clinical application of NfL measure in the context of the differential diagnosis of dementia [27]. However, the overlap of blood NfL levels between elderly patients with MDD and patients with mild cognitive impairment or AD is substantial (AUC = 0.50–0.57) [27].

When looking at adults with current depressive episodes, most studies reported elevated NfL levels in serum and plasma in patients with MDD compared to healthy controls (range: *n* = 24–94; 1.3- to 2.8-fold increase) (Table 1) [21, 87–92]. Positive correlations were found between NfL levels, cognitive dysfunctions (i.e. processing speed and executive functions), and white matter alterations, hinting at NfL elevation being associated with clinically relevant brain pathology [21, 87, 91]. While NfL findings in adults with recurrent MDD and ongoing depressive episodes have been quite consistent across studies, more contrasting results have been reported in young patients with untreated MDD, and in elderly patients with treatment-resistant or remitted MDD [92–95]. These observations might suggest a state dependent involvement of NfL alterations in MDD. However, the heterogeneity of clinical states considered in NfL studies on MDD, the use of unvalidated and potentially unreliable immunoassay methods in some studies [87, 90, 92], and the inconsistency in the confounding factors considered [21], prevent from drawing final conclusions on NfL involvement in the course of MDD. Moreover, the impact of antidepressant treatment on NfL levels remains to be elucidated through controlled longitudinal studies.

Intriguingly, blood NfL levels have also been associated with depressive symptoms in a broad spectrum of neuropsychiatric conditions and not exclusively in patients with MDD alone. In particular, elevated NfL levels have been reported in patients with secondary depressive symptoms linked to neurological disorders, such as stroke, TBI, MS, and PD, and in patients with comorbid MDD and SUD [93, 96–99]. Finally, serum NfL levels have been reported to be associated with depressive symptoms in the general population based on data from the 2013–2014 U.S. National Health and Nutrition Examination Survey but the lack of a more detailed clinical characterization in this sample limits the generalizability of these findings [100].

Relevant clinical applications of NfL measure in MDD have been suggested in the prediction and monitoring of treatment effects. NfL levels were found to be positively associated with treatment response following low-dose ketamine infusion [92]. Pilot studies on NfL in CSF (*n* = 9) and serum (*n* = 15) after electroconvulsive treatment, did not found alterations of NfL levels after the termination of a treatment session, thus supporting the long-term safety of this intervention [95, 101]. However, Hviid et al. did not find associations between NfL levels and clinical scores in a longitudinal investigation on MDD patients undergoing treatment with aerobic exercise or stretching [94].

Overall, current data suggest an elevation of blood NfL levels linked to current MDD (up to threefold increase) at lower magnitude compared to florid brain damage in neurological disorders such as FTD (up to tenfold increase) but significantly higher than in physiological aging (1.01- to 1.05-fold increase per year). Potential clinical applications of NfL for MDD have been suggested but only scarcely assessed so far.

### Bipolar disorder

Bipolar disorder (BD) have been linked to heterogeneous alterations of cortical and subcortical brain areas involving both gray and white matter structures. However, the regional involvement and the longitudinal course of structural brain changes in respect to manic/depressive episodes are inconsistent [2, 102–104].

Similarly to depression, serum NfL levels in small samples (range: *n* = 8–12) of elderly patients with BD were demonstrated to be significantly lower than levels in patients with neurodegenerative disorders, and rather comparable to HC [84, 85, 105]. Most investigations on NfL in adults and adolescent with BD reported elevated NfL levels in CSF, plasma, and serum (*n* = 25–133; 1.2- to 2.5-fold increase) compared to HC (Table 2) [22, 88, 90, 106–109]. In single studies, blood NfL levels were found to be associated with decreased cognitive performance and white matter alterations [90, 106, 110]. On the contrary, no alteration of CSF NfL levels were reported in a sample of adult patients with BD at both baseline and 1-year follow-up compared to HC [108]. In this sample, a trend level increase of plasma NfL levels were observed at baseline (1.19-fold increase) but not at follow-up.

Despite mostly supporting the view of elevated NfL levels in adults and adolescent with current BD, reports were so far highly heterogeneous in the clinical states considered (i.e., early vs. chronic, current manic episode vs. depressive episode vs. remitted, treated vs. untreated). Thus, the relationship between NfL levels and the clinical course of BD also considering potential differences between manic/depressive episodes remains unclear. Methodological inconsistencies in the use of immunoassay methods [90, 107] and in the characterization of confounding factors across studies limit final conclusions.

### Psychotic disorders

The role of structural brain alterations and neurodegeneration in the course of schizophrenia is controversial. According to the neurodevelopmental hypothesis, early alterations of brain structure predispose to later psychosis onset [111]. However, the presence of additional and progressive brain alterations occurring after the onset of first psychotic symptoms is still an issue of debate [112]. High structural brain heterogeneity was frequently reported in imaging studies [113].

Similarly to MDD and BD, the first investigations on NfL in patients with psychotic disorders were limited to small samples (range: *n* = 9–17) of elderly patients with the focus on the differential diagnosis of neurodegenerative disorders. NfL levels in blood and CSF of elderly patients with chronic schizophrenia were found to be comparable to HC and significantly lower than levels in neurodegenerative disorders [68, 84, 85, 105].

In adults with chronic schizophrenia, most studies reported no difference of mean NfL levels compared to HC in both CSF and blood (Table 3) [21, 88, 114, 115]. Rodrigues-Amorin described an elevation of plasma NfL levels in a subgroup of schizophrenia patients using clozapine, but this association was not confirmed in other studies [21]. Importantly, we demonstrated high heterogeneity of serum NfL levels in adults with clinically stable schizophrenia, with an increased proportion of patients with NfL levels above the 95th and 99th percentile of age-specific distribution curves and single subjects showing up to fourfold elevation NfL levels [21]. Higher variance of blood NfL levels in schizophrenia compared to HC were also observed in other studies but the limited sample size of the investigations so far does not allow a clinical characterization of subgroups based on NfL levels [114, 115].

Looking at early stages, Guasp et al. observed higher serum NfL levels in patients with first episode psychosis (FEP) compared to HC (1.39-fold increase with median age of 20 years) [116]. Similarly, Ceylan et al. reported a substantial elevation of serum NfL levels in children and adolescent with early onset schizophrenia (mean age 16 years) compared to HC (2.67-fold increase) [107]. These observations are particularly relevant, as the time window between childhood and early adulthood is considered critical for the occurrence of a putative “second hit” in the development of schizophrenia [117].

Finally, NfL has been suggested to find clinical application in the identification of secondary psychotic disorders induced by neurological conditions. In particular, Guasp et al. demonstrated that patients with anti–NMDA receptor encephalitis (NMDARe) present much higher serum NfL levels compared to patients with FEP (3.87-fold increase) [116]. Using a cutoff of serum NfL ≥ 15 pg/ml, the author demonstrated that 96% of patients with FEP and 85% of patients with NMDARe with isolated psychosis were correctly classified. Considering the challenge of identifying secondary immune psychosis from PPD in clinical settings, NfL measure could support clinical algorithms in patients with FEP of unclear etiology by identifying patients who should receive CSF antibody testing.

### Substance use disorders

The impact of SUD on structural brain integrity may depend on the substance considered, on the dose and exposure period, and on comorbid psychiatric disorders. The sensitivity of neurofilaments to substance-induced brain toxicity has been initially demonstrated in preclinical models including animal and in-vitro studies using cocaine, ketamine, 3,4-methylenedioxymethamphetamine (MDMA), and opiates [118–120]. In humans, elevated serum NfL levels were firstly reported in patients with ketamine dependence (KD) and heavy ketamine use (mean daily ketamine dose of 4.5 g) compared to HC (2.07-fold increase) [25]. Intriguingly, NfL levels in the KD group were higher in patients with history of MDD, suggesting potential mutual reinforcing effects of MDD in facilitating ketamine-induced brain pathology [93]. The interactive effects of the comorbidity of MDD and KD were later demonstrated in an additional study, where serum NfL levels were confirmed to be higher in patients with KD and MDD compared to patients with MDD or KD alone [93].

In a longitudinal study in individuals with cocaine use disorder, plasma NfL levels were reported to be increased at both baseline and 4-months-follow-up (1.39- and 1.54-fold increase, respectively) and to be positively associated with objective measures of cocaine use (hair cocaine concentration) [24]. In the same study, changes of NfL levels at follow-up were longitudinally predicted by changes of cocaine use in the interval time, confirming a dose-response relationship between cocaine use and NfL elevation.

When looking at the class of empathogens, Zimmerman et al. reported no alteration of serum NfL levels in chronic MDMA users [64]. Accordingly, diffusion tensor imaging confirmed that white matter integrity was not reduced in MDMA users in the same sample [64].

In patients with alcohol use disorder (AUD), Li et al. reported a strong elevation of serum NfL levels in AUD compared to HC (2.53-fold increase) [121]. Here, NfL levels were positively associated with NfL the degree of white matter lesions and negatively associated with global cognitive state (Montreal cognitive assessment score) and white matter volume. The authors also reported a positive association between reductions of alcohol use and normalization of NfL levels in a later follow-up measure. Clergue-Duval et al. observed higher plasma NfL in patients with severe AUD at first day of hospitalization for alcohol cessation compared to patients with at least 3 months of abstinence (1.59-fold increase) [122]. Integrating evidence from preclinical investigations, the authors suggested that NfL elevation may be driven by acute alcohol withdrawal rather than chronic alcohol use itself. Karoly et al. also found negative associations between plasma NfL levels and cortical thickness in heavy drinkers, but no comparison with HC was included [123].

Finally, in a study addressing mixed SUD, Requena-Ocaña et al. reported higher plasma NfL concentrations compared to HC and positive associations between NfL levels and dysfunctions in different cognitive domains [124]. However, the polydrug consumption patterns and the comorbidity with several psychiatric disorders makes it difficult to discern the contributions of single substances and different psychiatric conditions on NfL elevation in this study.

Taken together, consistent findings support the sensitivity of blood NfL measure to substance-related or -induced brain pathology and suggest intriguing applications for longitudinal monitoring (Table 4).

### Other psychiatric disorders

Two cross-sectional studies reported elevated blood NfL levels in children with autism spectrum disorders compared to typically developed children (1.44-fold increase with mean age 5.1 year; 1.93-fold increase with mean age 7.0 years) but no difference was seen in a third study (mean age 10 years) [125–127]. Notably, inconsistencies in use of immunoassay technology might partially explain the discrepant findings, taken that the latter study did not use fourth-generation immunoassay methods [127]. Data in adults with autism spectrum disorders are lacking so far.

In AN, two independent studies showed elevated blood NfL levels in patients with acute AN and underweight state (predominantly female adolescents) compared to HC (1.95-fold increase with mean age 16.4 years and 1.68-fold increase with mean age 26.0 years), which normalized after weight recovery [26, 128]. Elevated blood NfL levels in AN patients were also associated with lower cortical thickness in several brain regions with main clusters located in bilateral temporal areas [129]. Assessing patients with adolescent onset AN 30 years later (mean age of 44 years), Wentz et al. found increased serum NfL levels in patients compared to HC (1.46-fold increase) [130]. On the contrary, Doose et al. did not detect any differences in serum NfL levels in long-term weight-recovered women with a history of AN [131]. Nonetheless, lower BMI have been associated with increased NfL levels even in healthy individuals and this could clearly influence NfL levels in acute AN states independently of brain pathology [58].

Hansson et al. showed increased plasma NfL levels in first months of stress-related exhaustion (1.1-fold increase), with long-term normalization observed at follow-up (7–12 years later) [132]. On the contrary, Wallensten et al. detected no difference in plasma NfL levels in patients with stress-related mental disorders compared to HC, but analyzed a much smaller sample than Hansson et al. did (*n* = 31 vs. *n* = 150), and did not use highly sensitive immunoassay methods [133]. Potential associations between blood NfL levels and PTSD are limited to reports in survivor of mass violence or blast explosions, where TBI may be involved, which probably drives the NfL elevation [99, 134, 135].

Overall, discrepant findings in these less studied psychiatric conditions warrant standardized study settings, application of high sensitive fourth-generation assays, and correction for demographical and clinical factors known to affect the NfL concentration (Table 5).

### Secondary conditions with psychiatric symptoms

Associations between psychiatric symptoms and NfL levels have been also reported in a number of neurological (e.g., MS, PD, AD, stroke) [96–98, 136], inherited (e.g. Wilson’s disease and Down Syndrome) [137–140], and systemic (e.g., COVID-19, systemic lupus erythematosus, HIV) [141–146] conditions. While a systematic review of these diseases would go beyond the scope of the current review, the topic might have relevant implications for the psychiatric field. In particular, speculations might arise on the neurobiological interaction between NfL levels and secondary psychiatric symptom manifestations, considering that NfL integrity could influence behavioral presentations via synaptic (de-) stabilization [35]. Clinically relevant applications for liaison psychiatrist (e.g., patient stratification), differential diagnosis, and prognostic assessment might also arise. However, these research questions require disorder-specific considerations dependent on the nature of the primary pathological processes and should consider their specific clinical algorithms going beyond a merely psychiatric perspective. A separate future work on the topic is warranted.

## Opportunities and pitfalls for clinical applications

**Fig. 4:**
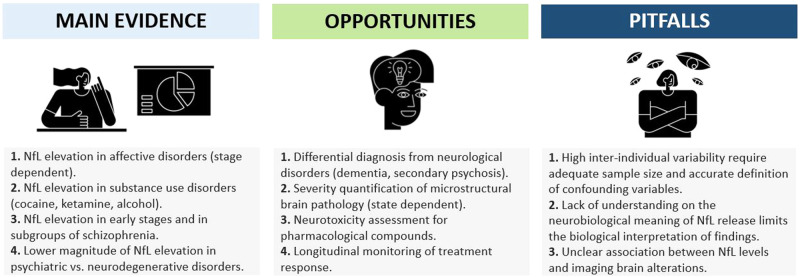
Main evidence, opportunities, and pitfalls for NfL use in psychiatry.

Current evidence convincingly shows that NfL levels might be affected by psychiatric disorders but also highlights high variability across individuals and clinical subgroups. Therefore, NfL measure in psychiatric conditions offers a window of opportunity for relevant clinical applications, but also poses some important challenges and pitfalls, that require critical consideration (Fig. 4).

### Diagnosis

Diagnostic markers in psychiatry are urgently needed and the introduction of blood NfL measure opens speculations on its potential use in diagnostic algorithms for psychiatric disorders. It is important to consider that NfL response to brain pathology is unspecific and NfL levels show high inter-individual variability even in healthy individuals (see section inter-individual variability below below) [9, 42]. This means that the degree of overlap of NfL levels in psychiatric and physiological conditions does not offer sufficient specificity for diagnostic definition of PPD based on single NfL measures. However, current evidence suggests that specific NfL cut-offs may effectively distinguish PPD from neurological conditions, with the most investigated application being the differential diagnosis of FTD with behavioral disturbances [68, 84, 85, 105]. The ability of NfL to distinguish AD from psychiatric disorders appears to be limited to early and rapidly progressive states, possibly because of the higher prevalence of cardiovascular risk factors in elderly individuals, which might obscure AD-related effects [27, 105]. Of relevance is also the use of NfL to identify autoimmune psychosis, such as NMDARe, where NfL cut-offs show good diagnostic performance in differentiating NMDARe from FEP [116]. Overall, age-dependent reference values for NfL may be used as screening measures to identify patients with high risk of having neurological conditions that would require mandatory follow-up diagnostic such us CSF sampling with autoantibody measure, or instrumental diagnostic (MRI, EEG). The increased variability of NfL levels in some psychiatric conditions may also open diagnostic possibilities toward better characterization of specific patients subgroups [21]. However, such data are still limited and larger cohort studies with transdiagnostic samples and comprehensive clinical assessment are needed.

### Severity assessment and prognosis

Associations between blood NfL levels and symptoms severity scores have been reported in MDD, where blood NfL levels were positively associated with cognitive dysfunctions (processing speed and executive functions) [21, 87]. NfL was also associated with the severity of self-reported depression in neurological disorders and in a population study [100]. In SUD, NfL levels were associated with self-reported and objectively assessed measures of substance intake and with the severity of substance use disorders [24, 121, 122]. The association between clinical severity and NfL levels in BD, schizophrenia is less clear [21, 22]. Importantly, state-dependent alterations of metabolic status (e.g., nutrition, BMI, hydration, renal function) might differently influence NfL levels in acute vs. chronic psychiatric conditions but no evidence on this topic is available so far. Whether NfL measure in blood or CSF may be used to stratify patients with psychiatric disorders based on the severity of structural brain involvement [129], or the possibility to use it for prognosis assessment remain to be elucidated [147]. The use of NfL levels to predict treatment response in MDD also represents an intriguing application but currently lacks sufficient clinical evidence [92].

In the case of SUD, particularly promising appears the use of NfL to compare the in vivo toxicity of different compounds. Toxicological hair analysis that objectively assess substance use combined with NfL measures may inform on the contribution of single illicit substances on structural brain pathology, thus offering a low-invasive tool to quantify substance-related neurotoxicity [24, 64]. However, larger studies including different substances and use patterns are needed.

### Longitudinal monitoring

Evidence from studies in neurological conditions clearly shows that blood NfL levels reflects state-dependent neuropathological processes rather than being a stable trait-marker of diagnostic entities [41]. Similarly, elevations of blood NfL levels in patients with MDD have been mostly shown in patients with current depressive episodes, but less in remitted patients with history of depression, thus supporting the view of state-dependent changes of NfL in MDD [21]. Increased blood NfL levels have been reported to be more prominent in early stages of schizophrenia or BD [107]. Normalization of NfL levels has been also described in chronic cocaine users after reduction of cocaine use [24], in patients with AUD after abstinence [148], and in patients with AN after clinical remission [128, 131]. Despite lacking any etiological specificity, these observations would support a clinical application of blood NfL measures as monitoring tool in psychiatric conditions, which would represent a great innovation in the field. The low degree of intra-individual fluctuations between timely closed consecutive NfL measures and the absence of diurnal variations of NfL levels are significant advantages in this regard [47, 48]. Thus, longitudinal assessment of blood NfL levels might be used to assess the safety and efficacy of therapeutic interventions. Considering the controversy on the putative neurotoxic/neuroprotective action of different psychiatric medications, blood NfL measure could provide a minimally-invasive, highly accessible tool to quantify the effects of pharmacological interventions on microstructural brain integrity [24, 95]. In addition, the response of NfL to a treatment intervention might guide the early identification of responders vs. non-responders [92, 148]. The current literature is insufficient to draw final conclusions, but the relevance of the topic strongly suggest the need for further investigations.

### Inter-individual variability and biological fluctuations

As stated above, NfL levels have a high degree of inter-individual variability even in healthy individuals and are influenced by a number of physiological and clinical factors that go beyond brain pathology. Age, BMI, renal function, head impacts, and cardiovascular risk factors are the main established confounding factors and should be assessed when planning an investigation on NfL [28, 41]. The impact of substance use (e.g., alcohol, cocaine, ketamine) on NfL levels has been demonstrated in patients with SUD but its relevance in occasional users is unclear [24, 64]. Age- and BMI-corrected reference values have been developed to at least partially overcome such limitations, but do not cover the entire physiological variability of NfL levels [58]. The potential influence of genetic and epigenetic predisposition on the inter-individual variability of NfL levels has been barely investigated so far [149]. Accordingly, future investigation on NfL in psychiatry should take into account main confounding factors and should include appropriate sample sizes to correct for inter-individual variability especially in cross-sectional studies. It is important to notice that metabolic and life-style factors may differ between patients with psychiatric conditions and thus elicit group-dependent effects on NfL levels, making the correction for such confounders particularly needed in psychiatry.

### Neurobiological correlates of NfL release in psychiatric dsiorders

The physiological processes underlying the release of NfL in peripheral matrices and the neuropathological alterations related to blood NfL increase in psychiatric conditions are still to be fully elucidated. On a microstructural level, membrane disintegration, axotomy, and axonal death have been clearly linked to extracellular release of NfL [150]. The determinants of NfL release in physiological and subclinical conditions in absence of evident axonal damage are still unclear. Intriguingly, inflammation has been postulated to play a pivotal role in the etiopathology of several psychiatric conditions and might be involved in NfL changes in psychiatric conditions [147, 151, 152]. However, clinical evidence on the association between inflammatory changes and NfL levels in PPD is still lacking [21]. Moreover, NfL levels in CSF and blood have been demonstrated to be robustly associated but the potential influence of blood-brain barrier permeability on NfL levels in psychiatric conditions is still to be elucidated [42, 44].

### Structural brain correlates of NfL alterations in psychiatry

In neurological conditions and healthy individuals, NfL levels have been associated with heterogeneous alterations of cortical thickness, gray matter volumes, white matter bundles and demyelination, as well as white matter intensities. Detailed reports on neuroimaging findings in specific neurological disorders can be find in previous publications [9, 10, 53, 153, 154]. In general, NfL levels were mostly correlated with neuroimaging markers of disease activity rather than specific neuroanatomic features, which is coherent with its ubiquitous distribution in the brain and its state-dependent elevation.

Data on the structural correlates of NfL levels in psychiatric conditions are limited yet. Two studies reported mixed associations between NfL levels and white matter integrity markers (i.e. fractional anisotropy and axial diffusivity) in affective disorders [91, 106]. Negative associations between NfL and white matter integrity markers (i.e. white matter diffusivity and volume) have been described in AUD [121, 123]. A single study reported state-dependent association between elevated NfL levels and decreased cortical thickness in AN [129]. Nonetheless, the different use of neuroimaging measures and immunoassay methods strongly limit the generalizability of the findings so far.

Importantly, the investigation of neuroimaging alterations linked to NfL elevations should take in account the clinical state and the time-frame of these markers. Macroscopic brain changes may be observed in remitted and clinically stable states, as consequence of cumulative brain damage in past disease episodes or pre-existing predisposition, while NfL seem to mainly reflect active brain pathology [71]. Therefore, longitudinal investigations including data on active episodes and, ideally, the assessment of NfL variations in repeated measures will be needed to clarify its impact on macroscopic brain structures.

## Conclusions: how to use NfL measure in psychiatry

**Fig. 5:**
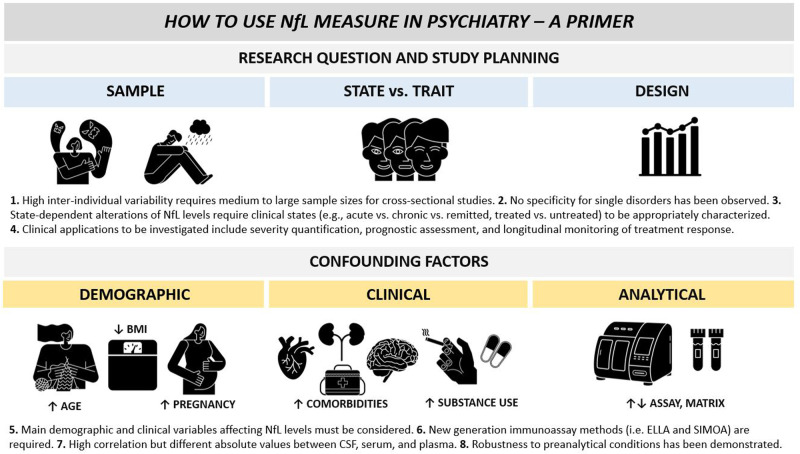
How to use NfL measure in psychiatry.

Blood NfL levels are altered in psychiatric disorders. While the extent of NfL alteration strongly vary across diagnostic entities, clinical stage, and patient subgroups, the magnitude of NfL elevation in patients with MDD, BD, AN, and SUD might reach a 1.2- to 2.5-fold increase. Despite lacking diagnostic specificity, these findings are remarkable, considering that physiological aging is associated with a nonlinear increase of NfL levels at a pace of only 1.01–1.05-fold per year [9, 18]. Accordingly, NfL measures offers intriguing opportunities for the implementation in psychiatry practice but some considerations are required for both study planning and data interpretation (Fig. 5). First, future studies should consider the relatively high inter-individual variability of NfL levels in both physiological and pathological conditions and the lack of specificity for single diagnostic entities. Second, NfL levels depend on clinical state and might longitudinally vary based on active biological processes. Clinical state variables should be appropriately characterized to find state-dependent associations with NfL levels. Accordingly, it might be preferable to consider different clinical stages separately (e.g., acute vs. chronic vs. remitted, treated vs. untreated). Third, NfL elevation above specific cut-off levels should elicit suspicion on an underlying neurological disease (e.g., FTD, PD, autoimmune psychosis) and should guide more in-depth instrumental diagnostic investigations. Fourth, moving beyond the framework of testing NfL for diagnostic purposes, NfL applications in PPD should take advantage of its suitability as state-dependent maker. Relevant opportunities might be found in the quantification of active brain pathology for subgroup stratification and prognostic assessment, in the assessment of brain toxicity for different pharmacological compounds, and in the longitudinal monitoring of treatment response. Finally, accurate characterization of demographic, clinical, and analytical confounding variables are necessary, as all these factors might strongly influence NfL levels. While some main confounding factors such as age, BMI, cardiovascular risk factors, and sport-related head impacts have been widely described, further research is needed to identify additional variables. Nonetheless, NfL offers unique advantages that make it truly innovative compared to other brain-derived markers including its robustness to preanalytical variations and the high degree of correlation between levels in blood and CSF matrices. Overall, the clinical application of NfL in psychiatry is an innovative and promising avenue that requires further investigations and might finding important applications in the clinical practice.
